# The Combined Effects of Fine Particulate Matter and Temperature on Preterm Birth in Seoul, 2010–2016

**DOI:** 10.3390/ijerph18041463

**Published:** 2021-02-04

**Authors:** Youngrin Kwag, Min-ho Kim, Shinhee Ye, Jongmin Oh, Gyeyoon Yim, Young Ju Kim, Eunji Kim, Semi Lee, Tai Kyung Koh, Eunhee Ha

**Affiliations:** 1 Department of Occupational and Environmental Medicine, Ewha Womans University, Seoul KS013, Korea; 2loveletter2@hanmail.net (Y.K.); kard141@naver.com (J.O.); olodii36@naver.com (E.K.); spring241226@gmail.com (S.L.); loueenie@naver.com (T.K.K.); 2 System Health & Engineering Major in Graduate School (BK21 Plus Program), Ewha Womans University, Seoul KS013, Korea; 3 Informatization Department, Ewha Womans University Seoul Hospital, Seoul KS013, Korea; mino-kim@naver.com; 4 Occupational Safety and Health Research Institute, Korea Occupational Safety and Health Agency, Incheon KS006, Korea; shinheeye88@gmail.com; 5 Department of Environmental Health, Harvard T. H. Chan School of Public Health, Boston, MA 02115, USA; hammer1219@naver.com; 6 Department of Obstetrics and Gynecology, College of Medicine, Ewha Womans University, Seoul KS013, Korea; kkyj@ewha.ac.kr

**Keywords:** preterm birth, low birth weight, PM_2.5_, temperature

## Abstract

**Background:** Preterm birth contributes to the morbidity and mortality of newborns and infants. Recent studies have shown that maternal exposure to particulate matter and extreme temperatures results in immune dysfunction, which can induce preterm birth. This study aimed to evaluate the association between fine particulate matter (PM_2.5_) exposure, temperature, and preterm birth in Seoul, Republic of Korea. **Methods:** We used 2010–2016 birth data from Seoul, obtained from the Korea National Statistical Office Microdata. PM_2.5_ concentration data from Seoul were generated through the Community Multiscale Air Quality (CMAQ) model. Seoul temperature data were collected from the Korea Meteorological Administration (KMA). The exposure period of PM_2.5_ and temperature were divided into the first (TR1), second (TR2), and third (TR3) trimesters of pregnancy. The mean PM_2.5_ concentration was used in units of ×10 µg/m^3^ and the mean temperature was divided into four categories based on quartiles. Logistic regression analyses were performed to evaluate the association between PM_2.5_ exposure and preterm birth, as well as the combined effects of PM_2.5_ exposure and temperature on preterm birth. **Result:** In a model that includes three trimesters of PM_2.5_ and temperature data as exposures, which assumes an interaction between PM_2.5_ and temperature in each trimester, the risk of preterm birth was positively associated with TR1 PM_2.5_ exposure among pregnant women exposed to relatively low mean temperatures (<3.4 °C) during TR1 (OR 1.134, 95% CI 1.061–1.213, *p* < 0.001). **Conclusions:** When we assumed the interaction between PM_2.5_ exposure and temperature exposure, PM_2.5_ exposure during TR1 increased the risk of preterm birth among pregnant women exposed to low temperatures during TR1. Pregnant women should be aware of the risk associated with combined exposure to particulate matter and low temperatures during TR1 to prevent preterm birth.

## 1. Introduction

Preterm birth is defined as labor and delivery at less than 37 gestational weeks. Preterm birth is associated with stillbirths and premature infant complications, resulting in substantial economic costs and impairments that persist into adulthood [1]. The high risk of morbidity and mortality among premature infants is partly attributable to growth restrictions associated with premature birth. In the long term, these conditions increase the risk of a variety of health hazards [2]. In Korea, birth outcomes among women of childbearing age are generally deteriorating [3]. This trend is maintained regardless throughout the childbearing years among women in Korea, where the low birth rate problem is serious [3].

According to a World Health Organization (WHO) report, maternal exposure to air pollution increases risk of preterm birth and low birth weight (LBW) (WHO, 2018). Fine particulate matter (PM_2.5_, or particles with an aerodynamic diameter of 2.5 µm or less) is more uniformly distributed in air compared with other pollutants. PM_2.5_ exposure is a useful indicator of overall exposure to outdoor particulate matter (PM) and can be used to study the health effects of such exposure [4]. Exposure to PM_2.5_ is strongly associated with adverse health effects [5]. Many studies have suggested that exposure to PM above a certain concentration affects the immune system [6]. PM_2.5_ enters the body and accumulates in various compartments of the respiratory tract, interacting with epithelial and immune cells [7]. Because the immune system is the most sensitive target of PM_2.5_, it is important to elucidate the impact of air pollutants on human immune function [8]. Inhaled PM_2.5_ can deposit in other compartments of the respiratory tract and interact with epithelial and immune cells. Consequentially, exposure to PM_2.5_ can cause local or systemic inflammatory responses [7].

Previous studies have reported that impaired immunity among pregnant women is associated with preterm birth [9,10]. Particulate matter and extreme temperatures increase risk of preterm birth by facilitating oxidative stress and interrupting the immune system. It has been suggested that inflammation and infection around the placenta are the potential biological mechanism underlying the association between air pollution and preterm birth [10].

The recent increase in environmental pollution, which is associated with high PM concentrations, has been shown to have negative effects on maternal immunity and neonatal health [11]. PM exposure may induce reproductive toxicity mediated by placental DNA methylation and maternal inflammatory responses, suggesting a link between PM exposure and preterm birth risk [12].

Changes in environmental temperatures may also affect maternal immune system [13]. Further, simultaneous exposure to PM and extreme temperatures during pregnancy may adversely impact immune function among pregnant women, which may trigger pregnancy complications [9,10]. For instance, several studies have reported that exposure to both PM and environmental temperature changes affect fetal growth and increase risk of premature birth or LBW [14,15]. However, few studies have investigated the combined effects of exposure to PM_2.5_ and ambient temperature on risk of preterm birth. To address this gap in the literature, the present study investigated the effect of prenatal exposure to PM_2.5_ in each trimester, as well as the combined effect of maternal PM_2.5_ exposure and temperature, on risk of preterm birth.

## 2. Materials and Methods

### 2.1. Birth Data

We used birth data from the MicroData Integrated Service (MDIS) system of the Korea National Statistical Office (https://mdis.kostat.go.kr). The MDIS system is a national statistical site containing sectorwide household and population data, including birth outcomes in the database.

A total of 605,026 babies were born from January 2010 through December 2016 in Seoul, Republic of Korea. After excluding the infants born (i) before 22 weeks or after 42 weeks of gestation and (ii) as part of multiple pregnancies (e.g., twins, triplets), 581,239 infants were included in our main analysis.

We used data from birth surveys, which included data on sex, birth weight, birth date, gestational age at birth, parity, season of birth, parental age, parental education status, parental occupations, and residential area.

### 2.2. Exposure Assessment

PM_2.5_ levels were measured using the Community Multiscale Air Quality (CMAQ) Modeling System [4]. Between 2009 and 2016, PM_2.5_ concentrations were estimated using the Weather Research and Forecast (WRF, version 3.3.1)—Sparse Matrix Operator Kernel Emission (SMOKE, version 3.1) Community Multiscale Air Quality (CMAQ, version 4.7.1) Modeling System [16]. We used the CMAQ data as PM_2.5_ exposure data because PM_2.5_ monitoring data has many missing values.

The CMAQ modeling data were generated using meteorological research and forecasting models comprising three overlapping weather data sources at 27, 9, and 3 km for a specific period of time [17]. The data are more representative of meteorological and atmospheric conditions than the Meteorological Agency’s monitoring data [18]. Additionally, the hourly CMAQ data have been collected at the administrative district level without any missing values. Daily data were calculated by averaging hourly CMAQ data over a 24-h period.

The temperature data from 2009 through 2016 were obtained from the Korea Meteorological Administration (KMA, www.kma.go.kr). The daily mean temperatures of each TR were divided into the following groups according to the quartile values: group 1 (−14.5 to 3.4 °C), group 2 (3.4–14.5 °C), group 3 (14.5–22.8 °C), and group 4 (22.8–31.8 °C).

### 2.3. Statistical Analysis

For the comparison between full-term and preterm babies, chi-square test was used for categorical variables, and Student’s *t*-test for continuous variables. Categorical variables are described as frequencies and percentages, and continuous variables are reported as means and standard deviations.

The mean PM_2.5_ and temperature exposure levels were calculated for each gestation period. During the prenatal period, each date was inversely estimated using gestational weeks provided in the microdata. PM_2.5_ data, temperature data, and birth data were integrated and analyzed using common local unit addresses and dates. The PM and temperature exposures were merged using the calculated dates and addresses, and the prenatal exposure periods were divided into trimesters based on the birth data and gestational age at birth to analyze the impact of exposure by period [19]: 1 to 12 weeks in the first trimester (TR1), 13 to 26 weeks in the second TR (TR2), and 27 to 40 weeks in the third TR (TR3).

In the analysis, the mean PM_2.5_ concentration was used in units of ×10 µg/m^3^ and temperature was analyzed in terms of quartiles: range 1 (−14.5 to 3.4 °C), range 2 (3.4–14.5 °C), range 3 (14.5–22.8 °C), and range 4 (22.8–31.8 °C).

Logistic regression models were used to evaluate the associations of prenatal exposure to PM_2.5_ and extreme temperature with risk of preterm birth. Model 1 included PM_2.5_ and temperature exposures of each trimester. Model 2 included PM_2.5_ and temperature exposures of all three trimesters and assumed the interactions between PM_2.5_ and temperature in each trimester. All models included child sex, mother’s age, mother’s educational level, parity, and season of birth as covariates. The models were calculated as follows:

Model 1: logit(π) = β0 + βTR1i + covariates. TRi: PM_2.5_ in the ith trimester, i = 1, 2, or 3.

Model 2: logit(π) = β0 + βTR11 + βTR22 + βTR33 + covariates. TR1: PM_2.5_ in the first trimester. TR2: PM_2.5_ in the second trimester. TR3: PM_2.5_ in the third trimester.

SAS version 9.4 (SAS Institute Inc., Cary, NC, USA) was used to perform all statistical analyses. The significance level was set at *p* < 0.05.

## 3. Results

The characteristics of the full-term and preterm births are presented in Table 1. Higher maternal age and lower maternal education were associated with higher preterm birth rates. Table 2 presents the quartiles of temperature and PM_2.5_ from March 2009 through February 2016 in Seoul.

The risk of preterm births by PM_2.5_ (10 µg/m^3^) exposure in each trimester is presented in Table 3. Model 1 does not assume the interaction between temperature and PM, and did not include exposure variables of other trimesters. The odds ratio of TR2 and TR3 in Model 2 indicates that an increase in PM exposure is associated with a decrease in the risk of preterm birth. However, in Model 2, which assumes the interaction and includes exposure variables of other trimesters, the maternal PM exposure in the TR1 only is significantly associated with an increased risk of preterm birth.

**Figure 1:**
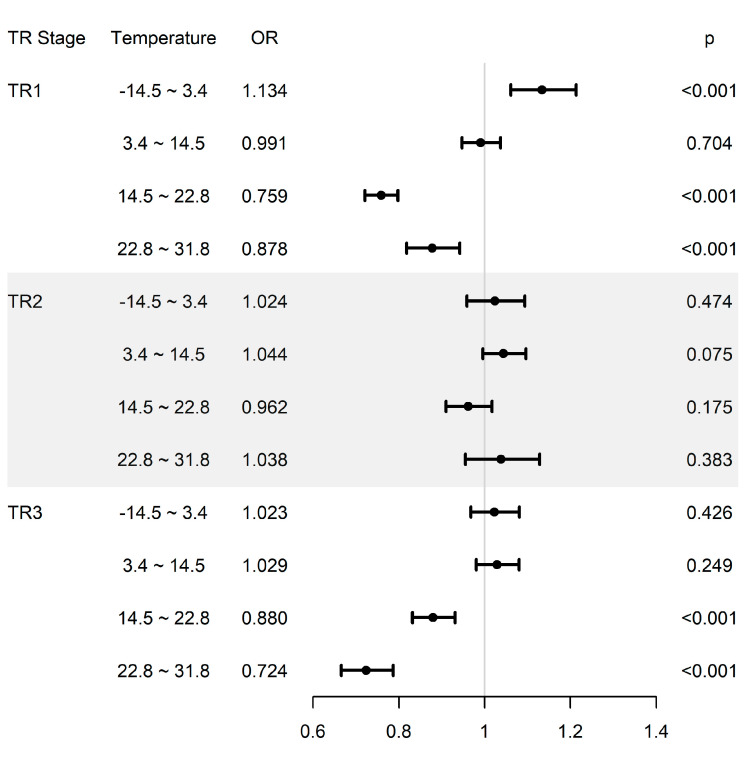
The combined effect of temperature (°C) and PM_2.5_ (×10 µg/m^3^) in each trimester on the risk of preterm birth. These results are based on the adjusted Model 2, including PM_2.5_ and temperature exposures of all three trimesters, and this results assume the interactions between PM_2.5_ and temperature in each trimester. The model adjusted for sex, mother’s age, mother’s educational level, parity, and birth season.

The risk of preterm birth due to the combined effect of extreme temperature and PM_2.5_ exposure is shown in Figure 1. In the model that mutually adjusted for each trimester, risk of preterm birth was highest during TR1 when the mean temperature during TR1 was less than 3.4 °C (OR 1.134, 95% CI 1.061–1.213, *p* < 0.001). On the other hand, the risk of preterm birth was lowest when the temperature was 14.5–22.8 °C during TR1.

## 4. Discussion

We found that the risk of preterm birth was associated with combined exposure to PM_2.5_ and low temperatures during TR1. 

According to a recent study, the risk of spontaneous preterm delivery increased following exposures to temperatures between 0 and −6 °C [20]. Temperature can have varying effects on adaptive immunity at multiple levels, with elevated temperatures inducing immune cell activation, function, and delivery, while reduced temperatures inhibit these processes [21]. Another study reported that exposure to air pollution at low temperatures induces complications associated with pregnancy [22]. The findings from these studies are consistent with our results. The impact of environmental extremes on the prevalence of preterm birth has been recognized [23]. Our results demonstrate that maternal prenatal exposure to extreme external temperatures can affect the timing of parturition.

Inhalation of PM_2.5_ particles induces oxidative stress and inflammation in pregnant women, which can contribute to the onset of preterm birth [24]. Air pollutants are complex substances that increase the risk of premature birth or LBW via inflammation, oxidative stress, endocrine disruption, and oxygen transportation disorders across the placenta [25]. As a result, exposure to air pollutants, such as PM_2.5_ during pregnancy, has also been associated with poor fetal growth [26]. Our results were consistent with the previous literature that TR1 is an important period of fetal development characterized by cell proliferation, invasion, and differentiation [27]. Regulation of immune responses at the placental interface occurs during early pregnancy; dysregulation during this time is the main driver of premature labor [28].

In our study, exposures between 22.8 and 31.8 °C were relatively lower than the range of high temperatures classically associated with premature birth. The high temperature range in Korea is 22.8–31.8 °C. The U-shaped dose–response relationship was observed between ambient temperature and risk of preterm birth in this study. Our results for high temperature exposure may be explained as follows. First, temperature represents the value of the average exposure temperature. Therefore, temperature exposure might not be constantly high, and the temperature may be lower than the data values presented. Second, the high temperature range consists of measurements higher than 30 °C. Third, in obstetrics and gynecology, “maintaining normothermia” refers to the comfortable and healthy environmental temperature ranging between 23 and 25 °C, which is associated with a lower risk of fetal death due to hypothermia. In our study, the range of 22–31 °C was relatively warm. In our study, if the temperature range was relatively narrow and the average exposure temperature was between 30 and 40 degrees, our results may be different.

It is important to explain not only the effect on premature birth of high temperatures but also the influence on premature birth of low temperatures. Many previous studies have demonstrated a U-shaped relationship between temperature and birth outcomes, reflecting a higher risk of premature births at extreme temperatures and a lower risk of premature births at moderate temperatures. Some relevant studies have focused on high temperatures above 40 °C. Our study’s main observation was that maternal exposure to low ambient temperatures during pregnancy could influence the timing of parturition. Additionally, we showed a protective effect of moderate ambient temperatures. Other studies have also reported that low temperatures increased the risk of preterm birth as much as high temperatures. Moderately warm temperatures have also been shown to be helpful for both the pregnant woman and her fetus [29], which is consistent with our findings. 

Our study accounted for both environmental epidemiological considerations and gynecological considerations. We also considered extreme temperatures on both ends of the scale. We investigated about 600,000 pregnancies from 2009 through 2016 using national data. This was not a cohort study observing detailed factors, but it was a study that comprehensively observed an association between human health and specific environmental determinants using national statistical data.

Since this was a retrospective study using national data, one limitation was that we were unable to control some factors that might have affected outcomes concurrently with the variables of interest. However, with this study of about 600,000 pregnant women from 2009 through 2016, we were able to comprehensively observe an association between human health and specific environmental determinants. Our study adjusted for sex, maternal age, maternal education level, parity, birth season, and temperature, but could not adjust for exposure in the working environment and outdoor activity time. Another limitation was related to the context of exposure assessment. Indoor temperatures and indoor PM_2.5_ exposure can be vastly different from their outdoor counterparts; the use of air conditioners and air purifiers indoors, for example, can contribute to such differences. These uncertainties about exposure settings should be considered when interpreting the study results.

## 5. Conclusions

We found that pregnant women exposed to low temperatures and PM_2.5_ during TR1 had a significantly increased risk of preterm delivery. This indicates that pregnant women should avoid very low temperatures and exposure to high concentrations of air pollutants simultaneously, particularly during TR1. Appropriate temperature control and fine particle management during TR1 could be an important measure to prevent preterm births. We need to consider not only extremely high temperatures but also the impact on premature births of extremely low temperatures.

## Figures and Tables

**Table 1:** Baseline characteristics of full-term and premature babies.

Characteristic		Full-Term (GA ≥ 37 Weeks)	Premature (GA < 37 Weeks)	*p*
		*n* (%) or Mean ± SD		
Sex	Male	283,774 (51.09)	14,616 (56.56)	<0.0001
	Female	271,625 (48.91)	11,224 (43.44)	
Birth weight (kg)		3.28 ± 0.38	2.43 ± 0.63	<0.0001
Gestational age at birth (weeks)		39.03 ± 1.07	34.35 ± 2.57	<0.0001
Parity of mother	1st	324,888 (58.61)	14,488 (56.67)	<0.0001
	2nd	193,259 (34.86)	9033 (35.33)	
	≥3rd	36,164 (6.52)	2043 (7.99)	
Season of birth	Spring	142,981 (25.74)	6300 (24.38)	<0.0001
	Summer	135,175 (24.34)	6740 (26.08)	
	Fall	137,980 (24.84)	6298 (24.37)	
	Winter	139,263 (25.07)	6502 (25.16)	
Age of mother (years)		31.90 ± 3.85	32.36 ± 4.22	<0.0001
Education level of mother	None	242 (0.04)	17 (0.07)	<0.0001
	Elementary	811 (0.15)	61 (0.24)	
	Middle	4646 (0.84)	315 (1.23)	
	High	96,978 (17.53)	5636 (21.98)	
	University	386,257 (69.82)	16,756 (65.34)	
	Graduate	64,266 (11.62)	2860 (11.15)	
Employment status of mother	No	295,870 (53.27)	14,393 (55.70)	<0.0001
	Yes	259,529 (46.73)	11,447 (44.30)	
Age of father (years)		34.38 ± 4.27	34.82 ± 4.57	<0.0001
Education level of father	None	187 (0.03)	12 (0.05)	<0.0001
	Elementary	788 (0.14)	56 (0.22)	
	Middle	4294 (0.78)	252 (1.01)	
	High	94,446 (17.20)	5194 (20.75)	
	University	367,083 (66.84)	16,037 (64.08)	
	Graduate	82,398 (15.00)	3477 (13.89)	
Employment status of father	No	18,164 (3.27)	905 (3.50)	0.0408
	Yes	537,235 (96.73)	24,935 (96.50)	

Abbreviation: GA, gestational age; SD, standard deviation.

**Table 2:** Quartiles of daily mean temperature (°C) and PM_2.5_ (µg/m^3^) in Seoul between 2009 and 2016.

	Min	Q1	Median	Q3	Max
Temperature (°C)	−14.5	3.4	14.5	22.8	31.8
PM_2.5_ (µg/m^3^)	3.5	18.5	27.2	37.9	200

Abbreviation: Min, minimum; Q1, first quartile; Q3, third quartile; Max, maximum.

**Table 3:** Risk of preterm births by PM_2.5_ (10 µg/m^3^) exposure in three trimesters.

Stage	Model 1 ^a^			Model 2 ^b^		
	OR	95% CI	*p*	OR	95% CI	*p*
TR1	0.965	0.920–1.012	0.143	1.134	1.061–1.213	<0.001
TR2	0.907	0.861–0.955	<0.001	1.024	0.959–1.094	0.474
TR3	0.947	0.902–0.993	0.025	1.023	0.968–1.081	0.426

Logistic regression models were used to calculate odds ratio (OR), 95% confidence interval (95% CI), and corresponding *p*-value. ^a^ Model 1 included PM_2.5_ and temperature exposures of each trimester. The model adjusted for sex, mother’s age, mother’s educational level, parity, and birth season. ^b^ Model 2 included PM_2.5_ and temperature exposures of all three trimesters and assumed the interactions between PM_2.5_ and temperature in each trimester. The model adjusted for sex, mother’s age, mother’s educational level, parity, and birth season.

## Data Availability

Not applicable.

## References

[1] Margerison-Zilko C.E., Li Y., Luo Z. (2017). Economic Conditions During Pregnancy and Adverse Birth Outcomes Among Singleton Live Births in the United States, 1990–2013. Am. J. Epidemiol..

[2] Abdallah Y., Namiiro F., Nankunda J., Mugalu J., Vaucher Y. (2018). Mortality among very low birth weight infants after hospital discharge in a low resource setting. BMC Pediatr..

[3] Ko H.S., Wie J.H., Choi S.K., Park I.Y., Park Y.G., Shin J.C. (2018). Multiple birth rates of Korea and fetal/neonatal/infant mortality in multiple gestation. PLoS ONE.

[4] Chang L.S., Cho A., Park H., Nam K., Kim D., Hong J.H., Song C.K. (2016). Human-model hybrid Korean air quality forecasting system. J. Air Waste Manag. Assoc..

[5] Gawda A., Majka G., Nowak B., Marcinkiewicz J. (2017). Air pollution, oxidative stress, and exacerbation of autoimmune diseases. Cent. Eur. J. Immunol..

[6] Zhao Q., Chen H., Yang T., Rui W., Liu F., Zhang F., Zhao Y., Ding W. (2016). Direct effects of airborne PM2.5 exposure on macrophage polarizations. Biochim. Biophys. Acta.

[7] Wei T., Tang M. (2018). Biological effects of airborne fine particulate matter PM. Environ. Toxicol. Pharmacol..

[8] Mikrut M., Regiel-Futyra A., Samek L., Macyk W., Stochel G., van Eldik R. (2018). Generation of hydroxyl radicals and singlet oxygen by particulate matter and its inorganic components. Environ. Pollut..

[9] Kim D., Chen Z., Zhou L.F., Huang S.X. (2018). Air pollutants and early origins of respiratory diseases. Chronic. Dis. Transl. Med..

[10] Gilman-Sachs A., Dambaeva S., Salazar Garcia M.D., Hussein Y., Kwak-Kim J., Beaman K. (2018). Inflammation induced preterm labor and birth. J. Reprod. Immunol..

[11] Hallman M., Haapalainen A., Huusko J.M., Karjalainen M.K., Zhang G., Muglia L.J., Rämet M. (2018). Spontaneous premature birth as a target of genomic research. Pediatr. Res..

[12] Cai J., Zhao Y., Liu P., Xia B., Zhu Q., Wang X., Song Q., Kan H., Zhang Y. (2017). Exposure to particulate air pollution during early pregnancy is associated with placental DNA methylation. Sci. Total Environ..

[13] Stower H. (2018). Predicting preterm birth. Nat. Med..

[14] Hartig T., Catalano R. (2013). Cold summer weather, constrained restoration, and very low birth weight in Sweden. Health Place.

[15] Arroyo V., Diza J., Carmona R., Ortiz C., Linares C. (2016). Impact of air pollution and temperature on adverse birth outcomes: Madrid, 2001–2009. Environ. Pollut..

[16] Han C., Kim S., Lim Y.H., Bae H.J., Hong Y.C. (2018). Spatial and Temporal Trends of Number of Deaths Attributable to Ambient PM. J. Korean Med. Sci..

[17] Leem J.H., Kim S.T., Kim H.C. (2015). Public-health impact of outdoor air pollution for 2(nd) air pollution management policy in Seoul metropolitan area, Korea. Ann. Occup. Environ. Med..

[18] Hakami A., Henze D.K., Seinfeld J.H., Singh K., Sandu A., Kim S., Byun D., Li Q. (2007). The adjoint of CMAQ. Environ. Sci. Technol..

[19] Suh Y.J., Kim H., Seo J.H., Park H., Kim Y.J., Hong Y.C., Ha E.H. (2009). Different effects of PM10 exposure on preterm birth by gestational period estimated from time-dependent survival analyses. Int. Arch. Occup. Environ. Health.

[20] Mathew S., Mathur D., Chang A.B., McDonald E., Singh G.R., Nur D., Gerritsen R. (2017). Examining the Effects of Ambient Temperature on Pre-Term Birth in Central Australia. Int. J. Environ. Res. Public Health.

[21] Appenheimer M.M., Evans S.S. (2018). Temperature and adaptive immunity. Handb. Clin. Neurol..

[22] Assibey-Mensah V., Christopher Glantz J., Hopke P.K., Jusko T.A., Thevenet-Morrison K., Chalupa D., Rich D.Q. (2019). Ambient wintertime particulate air pollution and hypertensive disorders of pregnancy in Monroe County, New York. Environ. Res..

[23] Mohammadi D., Naghshineh E., Sarsangi A., Zare Sakhvidi M.J. (2019). Environmental extreme temperature and daily preterm birth in Sabzevar, Iran: A time-series analysis. Environ. Health Prev. Med..

[24] Aceti A., Beghetti I., Martini S., Faldella G., Corvaglia L. (2018). Oxidative Stress and Necrotizing Enterocolitis: Pathogenetic Mechanisms, Opportunities for Intervention, and Role of Human Milk. Oxid. Med. Cell Longev..

[25] Bilbo S.D., Block C.L., Bolton J.L., Hanamsagar R., Tran P.K. (2018). Beyond infection-Maternal immune activation by environmental factors, microglial development, and relevance for autism spectrum disorders. Exp. Neurol..

[26] Michikawa T., Morokuma S., Fukushima K., Kato K., Nitta H., Yamazaki S. (2017). Maternal exposure to air pollutants during the first trimester and foetal growth in Japanese term infants. Environ. Pollut..

[27] Majali-Martinez A., Barth S., Lang U., Desoye G., Cervar-Zivkovic M. (2018). Temporal changes of the endothelin system in human cytotrophoblasts during the first trimester of pregnancy. Physiol. Res..

[28] Yadav A.K., Chaudhari H., Shah P.K., Madan T. (2016). Expression and localization of collectins in feto-maternal tissues of human first trimester spontaneous abortion and abortion prone mouse model. Immunobiology.

[29] He J.R., Liu Y., Xia X.Y., Ma W.J., Lin H.L., Kan H.D., Lu J.H., Feng Q., Mo W.J., Wang P. (2016). Ambient Temperature and the Risk of Preterm Birth in Guangzhou, China (2001–2011). Environ. Health Perspect..

